# Targeting Novel but Less Common Driver Mutations and Chromosomal Translocations in Advanced Non-Small Cell Lung Cancer

**DOI:** 10.3389/fonc.2017.00222

**Published:** 2017-09-29

**Authors:** Alia Daoud, Quincy S. Chu

**Affiliations:** ^1^Department of Medical Oncology, Cross Cancer Institute, University of Alberta, Edmonton, AB, Canada

**Keywords:** novel, non-small cell lung cancer, advanced, mutations, chromosomal rearrangement

## Abstract

Discovery of the epidermal growth factor receptor gene mutation and the anaplastic lymphoma kinase chromosomal translocation in non-small cell lung cancer has prompted efforts around the world to identify many less common targetable oncogenic drivers. Such concerted efforts have been variably successful in both non-squamous and squamous cell carcinomas of the lung. Some of the targeted therapies for these oncogenic drivers have received regulatory approval for clinical use, while others have modest clinical benefit. In this mini-review, several of these targets will be reviewed.

## Introduction

Epidermal growth factor receptor (EGFR) activating mutations in exons 18–21 and their exceptional responses to its kinase inhibitors (1, 2) marked the beginning of precision medicine in non-small cell lung cancer (NSCLC). Randomized trials showed treatment naïve, recurrent, or metastatic NSCLC patients harboring these mutations, particularly for exons 19 or 21 (3–10), had improved median progression-free survival (mPFS), tolerability, and quality-of-life from EGFR inhibitors over platinum-based chemotherapy. These studies triggered ongoing research to identify novel targets in both non-squamous (11, 12) and squamous NSCLC (13, 14) (Table 1). Crizotinib for ROS1 and dabrafenib/trametinib for BRAF mutation have received and submitted for regulatory approval, respectively. Selected targets, excluding EGFR and ALK, which will be discussed in separate reviews, will be discussed.

**Table 1:** Targets, mechanism(s) of target dysregulation, associated histology, and examples of current drugs in development and corresponding phase of clinical development in non-small cell lung cancer.

Target	Mechanism of target dysregulation	Histology associated	Example of targeted therapy	Phase of clinical development
BRAF	V600 Non-600	Adenocarcinoma	Dabrafenib/trametinib Vemurafenib ± cobimetinib LGX 818	Awaiting approval^a^ I/II I/II

DDR2	Mutation	Squamous	Dasatinib Nilotinib MGDC516	II II I/II

FGF1	Amplification	Squamous	Ponatinib AZD4547 BGJ 398 INCB054828 JNJ-42756493 TAS120 ARQ087 Debio 1347 E7090 LY287445	II I/II I/II I/II I/II I I I I I

HER-2	Exon 20 mutation HER-2 amplification	Adenocarcinoma	Afatinib Dacomitinib Trastuzumab ± pertuzumab T-DM1	II/III and approval^b^ II II II/III

K-RAS	Point mutation	Adenocarcinoma	MEK inhibition: Selumetinib trametinib CDK4/6 inhibitor: Palbociclib Abemaciclib Ribociclib	III I/II I/II II/III I/II I/II

MET	Amplification Exon 14	Non-squamous and squamous	Crizotinib Cabozantinib Foretinib Tepotinib Capmatinib Merestinib Volitinib Lorlatinib RXDX106 PLB001 HS10241	II-approval II II II I/II I/II I/II I/II/III I I I

NTRK	Translocation Point mutation	Adenocarcinoma	Entrectinib LOXO-101 AZD7451 DS 6051b MGCD516 PLX 7486 TPX00005	II I/II I I I I I

P3K/AKT/mTOR	PI3K mutation AKT mutation	Squamous cell carcinoma	PI3K inhibitor: Pan inhibitor: Buparlisib Copanlisib GSK2126458 MNL1117 XL147 CUDC-927 (HADC) PKB inhibitor: AZD8186 Alpelisib (BLY719) BGT 226 GDC0084 PI3K/mTOR inhibitor: BEZ 235 DS 7423 LY3023414 PF 04691502 VX-5584 XL 765 AKT inhibitor: Ipatasertib (GDC 0068) AZD 5363 GSK 2141795 LY2780301 Afuresertib ARQ 092 ARQ 751 BAY 1125976 ONC201 mTOR inhibitor: Temsirolimus Everolimus Vistusertib (AZD 2014) AZD 8055 BI 860585 CC-223 GDC 0349 ME-344 P70/S6K inhibitor: LY2584701 MSC 2363318A	II II II II I I I II I I I/II I I I I I II I/II I/II I/II I I I I I II II I/II I I I I I I I I I I I I I

RET	Translocation	Adenocarcinoma	Cabozantinib Lenvatinib Ponatinib Vandetanib BLU667	II II II II I

ROS1	Translocation	Adenocarcinoma	Crizotinib Cabozantinib Ceritinib Entrectinib Lolatinib DS 6015b TPX0005	Approval II II I/II II I I

*^a^Dabrafenib and trametinib combination has received approval from EMEA in February 2017 and has been submitted to the FDA for approval*.

*^b^Afatinib has regulatory approval for EGFR mutation positive, treatment naïve, advanced NSCLC, and previously treated squamous cell carcinoma by the FDA and EMEA*.

## BRAF Mutations

BRAF is a serine/threonine intracellular kinase and is activated by RAS, subsequently activates mitogen-activated protein kinase (MAPK). Activating BRAF mutations occur in 2–5% of NSCLC (15, 16). It is rare to find concurrent driver mutations, like K-RAS or EGFR (17). Activating BRAF mutations in NSCLC can be categorized into V600 and non-V600, in contrast to the predominance of V600 mutation in melanoma (15, 16). Although non-V600 BRAF mutations are more prevalent in heavy smokers, V600 mutants are found in never or light smokers (17). There are conflicting reports regarding the prognostic difference between the two subtypes (17).

BRAF inhibitors, such as vemurafenib, have shown promising preliminary benefit in V600 BRAF mutant, advanced NSCLC patients with a response rate (RR) of 42% and mPFS of 7.3 months (18). Planchard et al. (19) reported an RR of 33% and mPFS of 5.5 months with dabrafenib. Dual inhibition of BRAF and MEK with dabrafenib and trametinib yielded an RR of 63.2% and mPFS of 9.7 months in 57 evaluable patients (20). Dual inhibition prevents mechanisms leading to MAPK pathway reactivation (21, 22), resulting in more effective growth inhibition. However, resistant mechanisms to dual inhibition may arise as a result of RAS or ERK activation or mutation (23, 24), epigenetic EGFR alteration (25), or overexpression of MCL-1 (26). Non-V600 BRAF mutants may not be as responsive to BRAF inhibition based on BRAF-mutated melanoma data (15, 17, 20). There is no specific targeted therapy developed in this subpopulation.

## DDR2 Mutations

DDR2 is a receptor kinase that binds to collagen at the discoidin domain leading to its activation and subsequently to cell migration, proliferation, and survival (27, 28). Activating DDR2 mutations were identified in 4% of squamous NSCLC, with the majority in the kinase domain. Tumor growth inhibition by dasatinib was observed preclinically (29). One partial response (PR), in a patient with S768R DDR2 mutation and wild-type EGFR, of almost 1 year was reported in the Phase II trial of dasatinib and erlotinib in advanced NSCLC (30). Another PR was reported in the Phase II trial of dasatinib in previously treated, advanced NSCLC (31).

A Phase II trial of dasatinib in patients with either inactivating BRAF mutations or DDR2 mutations was conducted. It was terminated prematurely due to intolerable dyspnea, fatigue, and nausea. Patients were on therapy for 9–42 days, with no observed response (32). Trials of dasatinib and MGCD516 in DDR2 mutant solid tumors, including squamous NSCLC, are ongoing. Success in the development of DDR2 inhibitors should modulate the toxicity hindering adequate drug exposure and efficacy by careful dose and schedule selection.

## FGFR Pathway Aberrations

The FGF pathway consists of four receptors, FGFR1-4, and 18 ligands. Activation of the pathway leads to downstream activation of the RAS/RAF/MAPK, PI3K/AKT/mTOR, STAT, and PLCγ, which cause cell growth, proliferation, differentiation, migration, and survival (33). Pathway dysregulation can result from overexpression of either FGFs or their receptors, alternative splicing receptor isoforms, impaired downregulation, and degradation of activated FGF signal, FGFR gene amplification, point mutations, or chromosomal translocations (34). FGFR1 amplification is found in 10–25% of squamous NSCLC, commonly in smokers (35, 36). Whether FGFR1 amplification is prognostic remains controversial (36, 37).

An RR of 11.1% and disease-control rate (DCR) of 50% were reported in 36 FGFR1-amplified squamous NSCLC patients treated with BGJ398 (38). In the dose expansion cohort of the Phase 1 erdafitinib (JNJ-42756493) study, no response was observed (39). The criterion for FGFR1 amplification was not specified in either trial (38, 39). Two studies of AZD4547 reported 0/4 and 1/14 PR in evaluable FGFR1-amplified NSCLC, respectively. The responder had high FGFR1 amplification, defined as FGFR1:CEP8 ≥ 2.8 (40, 41).

It is premature to declare that FGFR1 amplification is not a driver mutation. Clinically significant toxicity from FGFR-targeted agents may occur at doses below which adequate growth inhibition of amplified FGFR1 tumors can be achieved. It is still unknown if FGFR1 amplification translates to overexpression or activation of the receptor. The definition of FGFR1 amplification needs to be refined, as in MET amplification and crizotinib efficacy (42).

Chandrani et al. reported that 5.5% of adenocarcinoma NSCLC harbor FGFR3 mutations at S249C, which was previously described in squamous NSCLC, and G691R which are sensitive to FGFR kinase inhibition in preclinical models. The clinical relevance will be established by future clinical trials (43).

## HER-2 Mutations and Amplification

HER-2 is a member of the EGFR family. The most common HER-2 mutation is exon 20 in-frame deletion or insertion between codons 776–779 (44, 45), which occur in 1.7–9% of all adenocarcinoma NSCLC (44–47). The length of insertion or deletion is heterogeneous (48). They are most commonly found in females and non-smokers. HER-2 exon 20 mutation or amplification leads to HER-2 phosphorylation, RAS/RAF/MAPK and PI3K/AKT/mTOR activation, and subsequent cell growth, proliferation, survival, and metastasis.

Six patients with HER-2 3+ or amplification had an RR of 83% and mPFS of 8.5 months as compared to an RR of 41% and mPFS of 7.0 months in those without after cisplatin/gemcitabine/trastuzumab treatment (49). A retrospective series of metastatic, HER-2 exon 20 mutant NSCLC reported DCRs of 93 and 100% after trastuzumab (*N* = 15) and afatinib (*N* = 3), respectively (46). A Phase II study of dacomitinib in 30 NSCLC with HER-2 aberrations reported an RR of 12% in those with exon 20 mutation and no response in those with amplification (50). The Phase II study of afatinib in 7 exon 20 mutant NSCLC had a DCR of 71% with 1 unconfirmed PR (uPR) (51). The ETOP NICHE trial of afatinib in HER-2 exon 20 mutant NSCLC reported a disappointing DCR at 12-week of 54% and mPFS of 13 weeks (52).

Phase II trials of ado-trastuzumab emtansine in HER-2 exon 20 or point mutations and HER-2 2+/3+ overexpressed NSCLC reported an RR of 6/18 and 10/49 with mPFS of 4 and 2.7 months, respectively (53, 54). The preliminary result of the ongoing MyPathway trial of trastuzumab and pertuzumab, targeting HER-2 dimerization, reported an ORR of 13 and 19% in 16 HER-2-amplified and 12-mutated NSCLC patients, respectively (55).

The benefit of HER-2-targeted therapeutics is modest. It is plausible that HER-2 exon 20 mutation and amplification represent two distinct molecular and therapeutic entities. There may be biological and therapeutic differences to HER-2-targeted agents based on the length of HER-2 exon 20 insertion or deletion (56). The full clinical and molecular data from these trials may help to elucidate the best treatment strategies to these subpopulations of HER-2 gene aberrant NSCLC.

## K-RAS Mutations

K-RAS is a member of the guanosine triphosphate gene superfamily. Upon activation by upstream receptors or point mutations at codons 12, 13, 14, or 60/61, K-RAS activates RAF/MAPK and PI3K/AKT/mTOR. These pathways regulate cell proliferation, growth, motility, and apoptosis (57).

K-RAS is mutated in 20–30% of NSCLC, predominantly in adenocarcinoma, non-Asians, and smokers. The incidence of K-RAS mutations may correlate with the amount of cigarettes smoked (11, 57). The majority of K-RAS mutations in NSCLC are at codon 12 (58). In a meta-analysis (59), K-RAS mutation was associated with poorer prognosis (HR = 1.45, 95% CI: 1.29–1.62), particularly in adenocarcinoma and early-stage NSCLC. It remains controversial if K-RAS mutation is predictive of platinum-based palliative chemotherapy efficacy (57), but it is associated with resistance to EGFR inhibitors. It is unclear if K-RAS mutation predicts efficacy to EGFR antibody (60–62), and if K-RAS transversion and transition mutations have different biology and thus therapeutic strategy and outcome (63, 64).

Targeting K-RAS mutation remains elusive. RAS attaches to the cell membrane for activation of downstream pathways *via* isoprenylation by farnesyltransferase. Alternatively, this is achieved by adding geranyl group by geranylgeranyltransferase I, particularly for K-RAS and H-RAS. Farnesyltransferase inhibitors failed possibly due to this geranylgeranyltransferase pathway (57, 65).

Current therapeutic approaches to K-RAS mutations in NSCLC focus on either the RAF/MAPK pathway or novel K-RAS biology. The MAPK pathway converges at MEK, which in turn activates ERK1/2. Targeting MEK will be expected to be effective in inhibiting the MAPK pathway, regardless of the upstream stimulatory signal. Despite encouraging Phase II results, the Phase III trial of docetaxel/selumetinib, an allosteric MEK1/2 inhibitor, combination over docetaxel alone in platinum-pretreated, advanced K-RAS mutant NSCLC (66), failed to confirm any survival improvement (67).

RAS activation drives G1/S cell cycle transition *via* cyclin-dependent kinase 2 and 4 (CDK2/4), induces cyclin D1, and downregulates the cdk inhibitor, p27KIP. Cycle D1 activates CDK4/6, which in turn phosphorylates retinoblastoma protein, leading to G1/S transition (68). K-ras mutant NSCLC animal models were particularly sensitive to CDK4/6 inhibition (69, 70). Synergistic antitumor activity was observed with trametinib and CDK4/6 inhibitor because MEK or ERK activation leads to cyclin D1 expression (71). A number of CDK4/6 inhibitors as single agent or in combination with MEK inhibitors are being studied in this population (72).

## MET Mutation and Amplification

MET is a receptor kinase and is activated by its ligand, hepatocyte growth factor, which plays a role in cell growth and development. It subsequently activates downstream RAS/RAF/MAPK, PI3K/AKT/mTOR, WNT/β-catenin, and STAT, promoting mitogenesis, motility, invasion, and morphogenesis (73, 74).

MET point mutation is detected in 3–4% of NSCLC. The most common is exon 14 splicing mutation (METex14) in 2–3% of NSCLC, who are older than 70 with non-squamous histology (sarcomatoid > adenosquamous and adenocarcinoma) and smokers. METex14 can have concurrent MET amplification, defined as MET/CEP7 ratio > 5.0 (75, 76). METex14 corresponds to the juxtamembrane domain, which is involved in its degradation by ubiquitin ligase, Cbl, leading to increase in MET activity (74, 77). METex14 alteration is highly variable, making it different to diagnose and predict therapeutic benefit (78). There has been encouraging preliminary antitumor activity of MET inhibitors in METex14 NSCLC (74), like an RR of 44 and 28% uPR after crizotinib (79).

It is challenging to define MET amplification. A recent study suggested the MET/CEP7 ratio > 5 as a sensitive and specific diagnostic test for MET amplification with low oncogenic driver overlap and highly predictive of crizotinib efficacy. These patients were mainly female and ex-smoker. High MET gene copy number was identified in 33% of adenocarcinoma NSCLC, however, none responded to MET inhibitor (80). The Phase II study of crizotinib in advanced NSCLC harboring MET amplification reported RR in low (>1.8–<2.2), intermediate (>2.2–<5) and high (>5) MET/CEP7 ratios of 0, 20, and 50%, respectively (42). It is important to determine MET amplification in non-responding EGFR mutants to EGFR inhibitors, as 2% of them have concurrent MET amplification (81).

Clinical development of MET inhibitors in MET aberration positive and in combination with EGFR inhibitors in EGFR mutant NSCLC is ongoing. This latter strategy may delay the emergence of MET amplification and thus prolong clinical benefit to EGFR inhibitors. Caution should be exercised in patient selection. Onartuzumab, MET antibody, or ARQ-197, MET kinase inhibitor, combined with erlotinib failed to improve survival in either unselected or non-squamous NSCLC with or without wild-type EGFR (82–84). Exploratory analysis found EGFR mutants had a trend toward poorer survival with onartuzumab/erlotinib (82).

## NTRK Mutation and Chromosomal Translocation

The NTRK family kinases, NTRK1–3, are activated by ligands from neurotrophin growth factor family. They are involved in neuronal development (85, 86). They subsequently activate downstream PI3K/AKT/mTOR, RAS/RAF/MAPK, PLC-γ, and protein kinase C, leading to cell proliferation, survival, and growth (86, 87). In addition, NTRK overexpression is prognostic (85, 88, 89). NTRK activation can result from translocation of the NTRK kinase to a transcription factor. NTRK1, NTRK2, and NTRK3 translocations account for 3.5, 0.2–1, and 1%, respectively, of adenocarcinoma NSCLC (87). NTRK1 and NTRK2 mutations were identified primarily in large cell carcinoma (85, 87).

Due to the structural similarity in the kinase domain of NTRK, ROS1, and ALK, several pan-inhibitors, such as entrectinib, LOXO101, and TPX-0005, are in clinical investigation. Initial Phase 1 studies reported encouraging preliminary antitumor activity and tolerability (87, 90). Identifying the primary and secondary resistant mechanisms, based on the understanding from ALK and ROS1, will help to improve the efficacy of current inhibitors and identify novel therapeutics, not limited to NTRK inhibitors targeting gatekeeper or solvent front mutation.

## PIK3CA/AKT/Phosphatase and Tension Homolog (pTEN)/mTOR Pathway Gene Aberrations

The PI3K/AKT/mTOR pathway is often activated in human cancers, leading to tumor proliferation, growth, and survival (91–93). There are three classes of PI3K. PIK3CA are heterodimers of a single p85 regulatory subunit, and one of the four isoforms of p110 catalytic subunits (α, β, γ, and δ). Different p110 subunit is preferentially expressed in different normal and malignant tissues. PIK3CA can be activated by upstream growth factor receptors, followed by AKT/mTORC1/p70S6K, which exerts a negative feedback on activated PIK3CA. In addition, tumor suppressor pTEN is a key negative regulator to PI3K/AKT/mTOR activation at PIK3CA (91, 94).

Several PI3K pathway activation mechanisms have been documented in NSCLC. Activating mutations in the exon 9 helical and exon 20 kinase domains are uncommon (92, 93, 95, 96). Amplification or polysomy is the predominant mechanism (92, 93). PIK3CA genetic alterations are thought to be more pivotal in squamous NSCLC pathogenesis. A study screening NSCLC, SCLC, extrapulmonary small cell cancer cell lines, and resected NSCLC identified PIK3CA gain in 33.1 and 6.2% of squamous and adenocarcinoma, respectively (92). Squamous NSCLC with PI3K family gene aberrations had inferior median overall survival (mOS) (8.5 versus 19.1 months, *p* < 0.0001), higher incidence of brain metastases, especially those with truncated pTEN loss (27 versus 11%, *p* < 0.0001), higher overall disease burden and genomic heterogeneity between the metastatic and primary tumors (37).

AKT consists of three isoforms, AKT1–3. Activating mutation, E17K in exon 4 kinase domain, accounts for 1–7% of all NSCLC (97, 98) with the majority being squamous NSCLC (99). Loss of pTEN expression occurred in up to 75% of NSCLC either by allelic loss (10–20%) (100, 101) or gene methylation (100, 102). It is postulated that pTEN loss leads to PIK3CAβ and downstream pathway activations.

Therapeutics targeting this pathway are currently in progress. Preliminary single agent antitumor activity has been disappointing. Toxicity, including hyperglycemia and GI toxicity, at least in part, limits the delivery of the optimal dose or schedule and thus antitumor activity. Inhibition of specific PIK3CA or AKT isoform leads to compensatory activation of other isoforms, limiting the antitumor activity. Due to extensive negative feedback loops, inhibition of a component leads to rebound activation of the pathway upstream (103). Ongoing studies to fully understand how to best target these genetic alterations, particularly in squamous NSCLC, with single agents, such as the LUNG MAP trial, or in combination with other complementary pathways, such as EGFR, HER-2, BRAF, may help optimize their efficacy.

## RET Chromosomal Translocation

RET is a kinase receptor for the giant cell-derived neurotrophic factor ligand. Binding of ligand leads to activation of RAS/RAF/MAPK, PI3K/AKT/mTOR, and PLC-γ, which regulate cell proliferation, migration, and differentiation. RET is important for renal organogenesis and enteric nervous system development (104).

RET was first determined to be oncogenic through the identification of interchromosomal translocation or intrachromosomal inversion in papillary thyroid cancer (105). Subsequently, RET chromosomal rearrangement was identified in NSCLC. The most common 5′ partner of the fusion oncogene is kinesin family member 5B, which is translocated to the kinase domain, leading to activation (106–110).

RET translocation is reported in 1–2% of NSCLC samples and are usually younger than 60, non-smoker, equally distributed in males and females and in mixed or solid adenocarcinoma. Over 30% have signet ring features (106–110).

Preliminary antitumor activity in Phase II trials with cabozantinib (111) and vandetanib (112, 113) demonstrated an RR of 18–47% and mPFS of 4.5–8 months. A global RET inhibitors registry reported an RR of 26% and mPFS of 2.3 months (114). The modest benefit from these multitargeted RET inhibitors may be related to subtherapeutic RET inhibition due to toxicity arising from inhibition of other targets. The heterogeneity of RET fusion partners and concurrent driver mutations may also impact the sensitivity to RET inhibitors. Highly selective RET inhibitors and better understanding of the biological differences in the fusion partners and concurrent mutations may help to improve the outcome of this NSCLC subtype.

## ROS-1 Chromosomal Translocation

ROS is a kinase receptor in the insulin receptor superfamily. Rearrangement occurs in 1–2% of non-squamous NSCLC (115, 116). ROS-1 chromosomal rearrangement leads to STAT3, PI3K/AKT/mTOR, and RAS/RAF/MAPK activation, followed by cell growth, proliferation, and survival (117). ROS-1 translocation NSCLC patient is described to be young, female, non-smoker, and with advanced stage adenocarcinoma (115, 117–120). The 5′ partners and the breakpoints of the ROS1 gene are variable (115, 116), which may impact on the biology and benefit to therapy.

The RR of 72%, mPFS of 19.2 months, and 1-year OS rate at 85% in 50 ROS-1 translocation NSCLC patients treated with crizotinib led to recent regulatory approval (121). Based on 77% homology in ALK and ROS-1, especially the kinase domain (121), ALK inhibitors are potentially efficacious. The Phase II study of ceritinib had an RR of 84% and mPFS of 19.3 months (122). In addition, pemetrexed-based chemotherapy may be effective, as ROS1 NSCLC have low thymidylate synthase mRNA levels (123). Further clinical validation is needed.

Overall, ROS1-rearranged NSCLC may have better prognosis with mOS of 36 months after standard chemotherapy and exceeding 5 years with chemotherapy and crizotinib. The incidence of brain metastases may be lower (123). Ongoing development of novel ROS1 inhibitors or combination to improve the benefit and to overcome resistance is important. It is conceivable that the resistant mechanisms to ROS1 inhibition parallel to those to ALK (124), such as secondary kinase domain mutations (125–127), which are sensitive to cabozantinib and lorlatinib, KIT mutation (128), RAS or EGFR pathway activation (129, 130).

## Conclusion

Multiple driver mutations have been identified in non-squamous and squamous NSCLC. There is regulatory approval of EGFR-, ALK-, ROS-1-, and BRAF-targeted agents. Benefits from therapies to other targets are preliminary.

To bring targeted therapeutics into the clinic, emphasis should be made on careful selection of true drivers. The criteria remain to be defined (131). Early clinical development efforts to identify and validate the most predictive biomarkers are key. With increasing number of driver mutations and therapies, and limited diagnostic tissues in advanced NSCLC, it is important to optimize diagnostic tissue accruement, minimize unnecessary pathological tests, and implement multiplex mutation analysis. The latter approach and basket trials, such as the LUNG MAP trial, exploring multiple targets simultaneously, can reduce the number and risk of biopsy, increase enrollment, and improve clinical trial efficiency.

Continual basic, translational, and clinical investigations are crucial to understand the targets, their resistance mechanisms, and corresponding therapies. For treatment tumor or plasma biopsies are necessary.

## Author Contributions

AD is an internal medicine rotating through medical oncology rotation. This manuscript served as one of her research projects. She was responsible to review the literature on a number of the therapeutic targets reviewed in the manuscript and provided her part of the corresponding manuscript. QC is the corresponding author who reviewed AD’s part of the manuscript, in addition to the review of other therapeutic targets in this manuscript.

## Conflict of Interest Statement

The authors declare that the research was conducted in the absence of any commercial or financial relationships that could be construed as a potential conflict of interest.
